# Supplementary material for: Coping strategy among the women with metastatic breast cancer attending a palliative care unit of a tertiary care hospital of Bangladesh
Source: PLoS One. 2023 Jan 13;18(1):e0278620. doi: 10.1371/journal.pone.0278620 (PMC9838864; doi:10.1371/journal.pone.0278620)
Supplement: S1 File — (DOCX) [file pone.0278620.s001.docx]

**Data collection sheet**

Project Title: **Coping Strategies among Women with Metastatic Breast Cancer attending Department of Palliative medicine, BSMMU**

Principal investigator: Dr. Nashid Islam

| Case No |  | Patient ID |  | Date of interview |  |
| --- | --- | --- | --- | --- | --- |

**Personal Information**

| **Name** | _____________________________________ | **Age** ______________________ |
| --- | --- | --- |
| **Education:** | **[1]** Can write name only  **[3]** High school (6-10)  **[5]** HSC  **[7]** Graduation  **[9]** PhD | **[2]** Primary level (1–5)  **[4]** SSC  **[8]** Post Graduation  **[10]** No Education |
| **Occupation** | **[1]** Housewife  **[3]** Govt. Service | **[2]** Private Service  **[4]** Business |
|  | **[5]** Unemployed | **[6]** Teacher |
|  | **[7]** Others ________________­ | **[8]** Healthcare Professionals |
| **Address:** | District: | Division: |
| **Religion** | **[1]** Islam **[2]** Hinduism**[3]** Christianity**[4]** Buddhism **[5]** Other | |
| **Marital Status** | **[1]** Unmarried **[2]** Married**[3]** Widow/Widower **[4]** Separated**[5]** Divorced | |
| **Phone** | Patient ____________________________ | Contact Person _______________________ |

**Clinical Information of patients with Metastatic Breast Cancer**

| **Symptoms at diagnosis:**  [1] Breast lump ( Right/ Left)  [2] Pain  [3] Discharge | [4] Other symptoms  A.  B.  C.  D. |
| --- | --- |

**Symptoms at present**

| [1] Pain | [2] Breathlessness | | [3] Cough |
| --- | --- | --- | --- |
| [4] Constipation | [5] Nausea and vomiting | | [6] ] Loss of appetite |
| [7] Bedsore | [8] Haemorrhage | | [9] Cachexia |
| [10] Weakness | [11] Itching | | [12] Weight loss |
| [13] Diarrhoea | [14] Drowsiness | | [15] Dry Mouth |
|  |  | |  |
| [16] Mouth Sore | [17] Fever | | [18] Others |
| [19] Swelling | [19.1] Upper Limb ( □ L / □ R / □ B) | [19.2] Lower Limb ( □ L / □ R / □ B) | |
|  | [19.3] Face | [19.4] Neck | |
|  | [19.5] Thorax | [19.6] Abdomen | |
|  | [19.7] Others___________________ |  | |

**Personal History**

[1] Smoking [2] Tobacco [7] None

[3] Betel leaf [4] Betel nut [8] others

[5] Alcohol [6] Drug dependency

**Socioeconomic Status**

Monthly Income (patient ± family) : _______________­­­­­_______ Taka

Total treatment Expenses upto now (according to patient /caregiver): ______________________ Taka

| **Expenditure borne by** | **[1]** Self | **[2]** Family |
| --- | --- | --- |
|  | **[3]** Loan | **[4]** Relatives |
|  | **[5]** Friends | **[6]** Community |

**Family History of Malignancy**

| **[1]** Yes (Relationship with patient) ________________ | | Disease ___________________________ |
| --- | --- | --- |
| **[2]** No |  |  |

**Physical sign:**

| [1] Anaemia  [2] Jaundice  [3] Ascites | [4] Oedema  [5] Ulcer  [6] Feeding Tube | [7] Catheter  [8] Examination of lump |
| --- | --- | --- |

[9] Neurological Deficit: Facial Palsy / Monoplegia/ Hemiplegia/ Paraplegia/ Quadriplegia

[10] Abdominalexamination: 1. Hepatomegaly2. Splenomegaly 3. Ascites

| [11] Lymphedema | [11.1] Upper Limb ( □ L / □ R / □ B) | [11.2] Lower Limb ( □ L / □ R / □ B) |
| --- | --- | --- |
| [12] Eastern Co-operative Oncology Group (ECOG) Performance Status | | |

| **Grade** | **Particular** | **Grade** | **Particular** |
| --- | --- | --- | --- |
| □ 0 | Asymptomatic | □ 3 | Symptomatic, >50% in bed, but not bed bound |
| □ 1 | Symptomatic but completely ambulant | □ 4 | Bed bound |
| □ 2 | Symptomatic, <50% in bed during the day |  |  |

**Primary Caregiver:**[1] Husband [2] Father [3] Mother [4] Sister [5] children [7] others

**Understanding of the patient**

| **[1]** Patient knows the diagnosis |  | **[1.a]** Yes | **[1.b]** No |
| --- | --- | --- | --- |
| **[2]** Patient understands the prognosis |  | **[2.a]** Yes | **[2.b]** No |

**Understanding of the family**

| **[1]** Family knows the diagnosis |  | **[1.a]** Yes | **[1.b]** No |
| --- | --- | --- | --- |
| **[2]** Family understands the prognosis |  | **[2.a]** Yes | **[2.b]** No |
| **[3]** Family accepts the prognosis. |  | **[3.a]** Yes | **[3.b]** No |

**Tumour Variable**

| **Metastasis** | **[1]** Brain | **[2]** Lung (L / R / B) |
| --- | --- | --- |
|  | **[3]** Liver | **[4]** Bone (site) ______________ |
|  | **[5]** Breast (L / R / B) | **[6]** Lymph node |
|  |  |  |
| **Histopathology** | [i] ______________________________ | Date: ___________________________ |
|  | [ii] _____________________________ | Date: ___________________________ |
|  | [iii] _____________________________ | Date: ___________________________ |
|  | **[] Not done** |  |
|  |  |  |
| **Hormone receptor** | [1] positive | [2] Negative |
|  |  |  |
| **Stage** | **[1]** 1 | **[2]** 2 |
|  | **[3]** 3 | **[4]** 4 |
|  | **[5]** Not Done |  |
| **Grade** | **[a]** Well Differentiated | **[b]** Moderately Differentiated |
|  | **[c]** Poorly Differentiated | **[d]** Undifferentiated |
|  | **[e]** Not Done |  |

**Associated Illness**

| **[1]** DM | **[2]** HTN | **[3]** IHD | **[10] None** |
| --- | --- | --- | --- |
| **[4]** COPD | **[5]** PUD | **[6]** TB |  |
| **[7]** Bronchial Asthma | **[8]** Stroke | **[9]** Others _____________ |  |

**Details of Treatment Received [1] Applicable[2] Not Applicable**

| **Radiotherapy** | **[1]** Yes | **[2]** No |
| --- | --- | --- |
|  |  |  |
|  | If yes, Schedule completed □ Yes □ No ________________________ | |
|  | **[3]** side effect ________________ |  |

| **Chemotherapy** | **[1]** Yes | **[2]** No |
| --- | --- | --- |
|  | If yes, Schedule completed □ Yes □ No ________________________ | |
|  | **[3]** side effect ________________ |  |

| **Biological / Immune therapy** | **[1]** Yes | **[2]** No |
| --- | --- | --- |
|  |  |  |
| **Targeted / Hormone therapy** | **[1]** Yes | **[2]** No |

| **Surgery** | **[1]** Yes | **[2]** No |
| --- | --- | --- |
|  | **[3]** Operation Name ______________**________________________________** | |

| **Consultation**: | [1] Surgeon  [2] Oncologist  [3] Radiotherapist | [4] Medicine specialist  [5] Psychiatrics  [6] Counselor |
| --- | --- | --- |

| **Treatment in Abroad.** | **[1]** Yes | **[2]** No |
| --- | --- | --- |

**Complementary Therapy**

| **[1]** Ayurvedic | **[2]** Unani | **[3]** Homeopathy |
| --- | --- | --- |
| **[4]** Religious approach | **[5]** Others_________________ | **[6]** None |

**Hospital Anxiety and Depression Scale (HADS)**

গত সপ্তাহের বিবেচনায় নিচের প্রশ্নগুলোর জন্য আপনার সবথেকে কাছের উত্তরটি বাছাই করুন।

| **D** | **A** |  | **D** | **A** |  |
| --- | --- | --- | --- | --- | --- |
|  |  | **আমি চিন্তিত অনুভব করি? *** |  |  | **আমি অনুভব করি, আমি আমার কাজে পূর্বের চেয়ে ভীষণ মন্থর হয়ে পরেছি? *** |
|  | 3 | বেশির ভাগ সময় | 3 |  | সবসময় |
|  | 2 | সব সময় | 2 |  | প্রায়শই |
|  | 1 | মাঝেমধ্যে | 1 |  | মাঝেমধ্যে |
|  | 0 | একদমই না | 0 |  | কখনোই না |
|  |  | **আমি বই পড়া, মুভি দেখা বা অন্য যে কোনো বিনোদনমূলক কাজ উপভোগ করি? *** |  |  | **আমি কিছুটা ভীতসন্ত্রস্ত হয়ে পড়ি আমার মনে হয় পেটে অস্বস্তি অনুভব করছি? *** |
| 0 |  | আগের মতোই |  | 0 | কখনোই না |
| 1 |  | আগের চেয়ে কম |  | 1 | খুবই কম |
| 2 |  | মাঝেমধ্যে |  | 2 | প্রায়শই |
| 3 |  | খুবই কম |  | 3 | সবসময় |
|  |  | **মাঝেমধ্যে মনে হয় আমার সাথে খারাপ কিছু হতে যাচ্ছে? *** |  |  | **আমি নিজের পোশাক-আশাক নির্বাচন ও সাজসজ্জার প্রতি অনাগ্রহ অনুভব করি? *** |
|  | 3 | খুবই খারাপ ভাবে মনে হয় | 3 |  | অনেকাংশে |
|  | 2 | মনে হয় কিন্তু তেমন বেশি না | 2 |  | আমি নিজের ততটা পরিচর্যা করি না যতটা করা উচিত বলে মনে করি। |
|  | 1 | খুবই কম | 1 |  | হয়তোবা আমি আমার নিজের যথেষ্ঠ পরিচর্যা করি না |
|  | 0 | একদমই না | 0 |  | আমি আমার নিজের যথেষ্ঠ পরিচর্যা করি |
|  |  | **আমি সব সময় হাসি খুশি থাকার চেষ্টা করি, এবং সব কিছুর ভালো দিক টা দেখি? *** |  |  | **আমি খুব সহজেই ভীষণ অস্থির এবং অধৈর্য হয়ে পরি যেহেতু আমাকে সবসময় ভীষণ তাড়াহুড়ো এবং কাজের চাপে থাকতে হয়? *** |
| 0 |  | সব সময় চেষ্টা করি |  | 3 | অনেক বেশি |
| 1 |  | এখন তেমন বেশি না |  | 2 | কিছুটা |
| 2 |  | মাঝেমধ্যে |  | 1 | খুব একটা না |
| 3 |  | একদমই না |  | 0 | একদমই না |
|  |  | **আমি সব সময় কোন না কোন বিষয় নিয়ে চিন্তা করতে থাকি? *** |  |  | **আমি উৎসাহের সাথে নতুন জিনিসের জন্যে অপেক্ষা করি? *** |
|  | 3 | সব সময়ই | 0 |  | পূর্বের মতই |
|  | 2 | প্রায় সব সময়ই | 1 |  | পূর্বের চেয়ে হয়ত কিছুটা কম |
|  | 1 | মাঝেমধ্যে | 2 |  | পূর্বের চেয়ে অনেক কম |
|  | 0 | খুবই কম | 3 |  | একদমই না |
|  |  | **আমি আনন্দিত অনুভব করি? *** |  |  | **আমি হঠাৎ করেই ভীষণ আতংকিত অনুভব করি? *** |
| 3 |  | একদমই না |  | 3 | অনেক বেশি |
| 2 |  | খুব কম |  | 2 | কিছুটা |
| 1 |  | মাঝেমধ্যে |  | 1 | খুব একটা না |
| 0 |  | সব সময় |  | 0 | একদমই না |
|  |  | **আমি সাচ্ছন্দ্যে/স্থির হয়ে বসে আরাম করতে পারি? *** |  |  | **আমি যেকোন একটি ভাল বই অথবা রেডিও কিংবা টেলিভিশনের যেকোন বিনোদনমূলক অনুষ্ঠান উপভোগ করতে পারি? *** |
|  | 0 | সব সময় | 0 |  | প্রায় সবসময় |
|  | 1 | প্রায়ই | 1 |  | মাঝেমধ্যে |
|  | 2 | খুব কম | 2 |  | খুব একটা না |
|  | 3 | একদমই না | 3 |  | না বললেই চলে |

**Appendix – 2(b):Bangla Version of the Brief-COPE**

**Original Developer: Carver (1997)**

**Translation and Validation in Bangla: Islam and Hossain (2020)**

**নিন্মে মানসিক চাপমূলক পরিস্থিতি মোকাবেলা ক**রার জন্য আমারা সাধারণত যে পন্থাগুলো অবলম্বন করি সে সম্পর্কিত কিছু উক্তি রয়েছে। আপনি **এ ধরনের পরিস্থিতিতে নিম্মোক্ত পন্থাগুলো কতটুকু অবলম্বন করেন তা ৫টি উত্তরের যে কোন একটিতে** টিক চিহ্ন (√) দিয়ে প্রকাশ করুন। অন্যরা এই পরিস্থিতিতে কি করে তা চিন্তা না করে আপনি কি করেন তা চিন্তা করে উত্তর দিন।

৫টি উত্তরঃ**১= একেবারেই প্রযোজ্য নয়, ২= প্রযোজ্য নয়, ৩= অনিশ্চিত, ৪= প্রযোজ্য, ৫= সম্পূর্ণ প্রযোজ্য**

এখানে**‘এ ধরনের পরিস্থিতিতে’** বলতে**‘মানসিক চাপমূলক পরিস্থিতিটিকে’** বুঝানো হয়েছে। সুতরাং উক্তির বোধগম্যতার সুবিধার্থে**‘এ ধরনের পরিস্থিতিতে’**এর স্থলে**‘মানসিক চাপমূলক পরিস্থিতিটি’** চিন্তা করবেনতাহলে উক্তিটি বুঝা সহজ হবে।

| উক্তিসমূহ | | | | | |
| --- | --- | --- | --- | --- | --- |
| ১। এ ধরনের পরিস্থিতিতে মনোযোগ না দিয়ে আমি নিজেকে অন্য কাজে ব্যস্ত রাখি। | ১ | ২ | ৩ | ৪ | ৫ |
| ২। আমি যে পরিস্থিতিতে থাকি সেখানে আমার সমস্ত প্রচেষ্টা দিয়ে কিছু করার চেষ্টা করি। | ১ | ২ | ৩ | ৪ | ৫ |
| ৩। এ ধরনের পরিস্থিতিতে আমি নিজেকে বুঝাই “এমন কিছু ঘটেনি”। | ১ | ২ | ৩ | ৪ | ৫ |
| ৪। আমি নিজে আরো ভালোবোধ করতে মদ অথবা অন্য ঔষধ গ্রহন করি। | ১ | ২ | ৩ | ৪ | ৫ |
| ৫। আমি অন্যদের কাছ থেকে মানসিক সমর্থন পেয়ে থাকি। | ১ | ২ | ৩ | ৪ | ৫ |
| ৬। আমি এ ধরনের পরিস্থিতি মোকাবেলা করার চেষ্টা থেকে বিরত থাকি। | ১ | ২ | ৩ | ৪ | ৫ |
| ৭। পরিস্থিতিটি ভালো করার জন্য আমি কিছু করার চেষ্টা করি। | ১ | ২ | ৩ | ৪ | ৫ |
| ৮। আমি বিশ্বাস করতে রাজি নই যে, এমন কিছু ঘটেছে। | ১ | ২ | ৩ | ৪ | ৫ |
| ৯। আমি আমার অপ্রীতিকর অনুভুতিগুলো থেকে মুক্তি পেতে অন্যের সাথে কথা বলি। | ১ | ২ | ৩ | ৪ | ৫ |
| ১০। আমি অন্যের কাছ থেকে সাহায্য ও পরামর্শ পেয়ে থাকি। | ১ | ২ | ৩ | ৪ | ৫ |
| ১১। আমি এ ধরনের পরিস্থিতি কাটিয়ে উঠতে মদ অথবা অন্য ঔষধ গ্রহন করি। | ১ | ২ | ৩ | ৪ | ৫ |
| ১২। আমি এ ধরনের পরিস্থিতিকে ভিন্নভাবে দেখার চেষ্টা করি যাতে এটি আরো বেশী ইতিবাচক মনে হয়। | ১ | ২ | ৩ | ৪ | ৫ |
| ১৩। আমি নিজের সমালোচনা করি। | ১ | ২ | ৩ | ৪ | ৫ |
| ১৪। এ ধরনের পরিস্থিতিতে কি করতে হবে তার জন্য আমি একটা উপায় বের করার চেষ্টা করি। | ১ | ২ | ৩ | ৪ | ৫ |
| ১৫। আমি অন্যের কাছ থেকে সান্ত্বনা এবং সহমর্মিতা পেয়ে থাকি। | ১ | ২ | ৩ | ৪ | ৫ |
| ১৬। আমি এ ধরনের পরিস্থিতি মোকাবেলা করার চেষ্টা পরিত্যাগ করি। | ১ | ২ | ৩ | ৪ | ৫ |
| ১৭। যা ঘটছে তার মধ্যে আমি ভালো কিছু খোঁজার চেষ্টা করি। | ১ | ২ | ৩ | ৪ | ৫ |
| ১৮। আমি এ ধরনের পরিস্থিতি নিয়ে রসিকতা করি। | ১ | ২ | ৩ | ৪ | ৫ |
| ১৯। এ ধরনের পরিস্থিতি নিয়ে যেন বেশি ভাবতে না হয় তার জন্য আমি এমন কিছু করি যেমনঃ সিনেমা দেখতে যাই, টিভি দেখি, পড়ি, অবাস্তব কল্পনা করি, ঘুমাই অথবা কেনাকাটা করি। | ১ | ২ | ৩ | ৪ | ৫ |
| ২০। যে ঘটনা ঘটেছে তার বাস্তবতা আমি মেনে নেই। | ১ | ২ | ৩ | ৪ | ৫ |
| ২১। আমি আমার খারাপ লাগার অনুভুতিগুলো প্রকাশ করি। | ১ | ২ | ৩ | ৪ | ৫ |
| ২২। আমি আমার ধর্মে অথবা ধর্মীয় বিশ্বাসে স্বস্তি খোঁজার চেষ্টা করি। | ১ | ২ | ৩ | ৪ | ৫ |
| ২৩। এ ধরনের পরিস্থিতিতে কি করতে হবে সে বিষয়ে আমি অন্যদের পরামর্শ অথবা সাহায্য নেয়ার চেষ্টা করি। | ১ | ২ | ৩ | ৪ | ৫ |
| ২৪। আমি এ ধরনের পরিস্থিতি মেনে নিতে জানি। | ১ | ২ | ৩ | ৪ | ৫ |
| ২৫। এ ধরনের পরিস্থিতিতে আমি গভীরভাবে চিন্তা করি কি পদক্ষেপ নেয়া যায়। | ১ | ২ | ৩ | ৪ | ৫ |
| ২৬। যা ঘটছে তার জন্য আমি নিজেকে দায়ী করি। | ১ | ২ | ৩ | ৪ | ৫ |
| ২৭। আমি প্রার্থনা করি অথবা চিন্তামগ্ন থাকি। | ১ | ২ | ৩ | ৪ | ৫ |
| ২৮। আমি এ ধরনের পরিস্থিতি নিয়ে মজা করি। | ১ | ২ | ৩ | ৪ | ৫ |
