# Supplementary material for: Coping strategy among the women with metastatic breast cancer attending a palliative care unit of a tertiary care hospital of Bangladesh
Source: PLoS One. 2023 Jan 13;18(1):e0278620. doi: 10.1371/journal.pone.0278620 (PMC9838864; doi:10.1371/journal.pone.0278620)
Supplement: Attachment — Submitted filename: PONE-D-22-12704_Review_09142022.pdf [file pone.0278620.s002.pdf]

Coping Strategy among the Women with Metastatic Breast Cancer Attending a Palliative Care Unit of a Tertiary Care Hospital of Bangladesh

REVIEW PLOS ONE

This is a very interesting topic which analyse adaptive behavior of women confronted with a metastatic breast cancer. This analyse could allow to help some patients to accept their disease, better apprehend and improve their quality of life during this step of their life. The methodology is clear and well thought out.

However, I believe certain matters need further clarification before publication.

Summary:

- What do the average of the different domains (acceptance, religion, emotional and instrumental support) correspond to? It's not specify in the summary's methodology.
- Can you write clearly what domain are correlated with each other? Are all these domains (emotional support, active coping, planning, acceptance, behavioral disengagement, venting) correlated with religion?
- In the last line of summary "and" is repeated 2 times: "and and management…"

Background:

- In line 64: Can you correct "Diagnosis of metastatic breast cancer it is a great shock". The correct phrase would be "Diagnosis of metastatic breast cancer is a great shock".
- In line 64 I would write the sentence like that: "Diagnosis of metastatic breast cancer is a great shock for the patient. Its treatment and side effects have tremendous social and psychological impacts on her.
- Line 68: You say: "In Bangladesh, 68 late presentation of breast cancer is very common mostly because of social stigma and lack 69 of awareness". So, are you sure of the percentage of metastatic first diagnosis (6%)? It seems low.

Methods:

Globally the methodology is well explained.

- Line 133: a point is missing after "questionnaire" and "it" just after should be deleted.

Results

- Line 172: "More than half of them (68.4%) had no family history of malignancy": I would add "history of malignancy known".
- Table 1: Mean of age is already notify in the text. It's not necessary to put it in the table. I would write there also "Family history of malignancy".
- Line 182: "patients" is repeated 2 times.

Discussion

- Line 224: Can you correct this phrase. It not grammatically correctly written. I would write "In our study women, suffering from metastatic breast cancer, most frequently used coping strategies like acceptance, religion, emotional support, instrumental support, and planning. But…"
- Line 226: Do you want to say "behavioral disengagement"?
- Line 268: Can you reformulate this phrase because it is not correct. Can specify seem to have what?
- Line 280 to 282: Can you separate in two sentences?
- Line 284: Paragraph from "One interesting finding…" You can't say patients with this ECOG develop or adopt such and such coping strategy, because you don't know what is the order of the mechanism. Namely, is the coping strategy a consequence of ECOG or is ECOG a consequence of coping strategy? You can only say that Patients with such and such ECOG have such and such coping strategy. The analysis only shows the correlation between both but don't specify what comes first.

Conclusion

- Line 296: Idem than paragraph line 284.
- Line 299: and is repeated two times.
